# Type I Atelocollagen Interposition Within the ACL Graft During ACL Reconstruction: An Exploratory Clinical and MRI-Based Study

**DOI:** 10.3390/medicina62061176

**Published:** 2026-06-17

**Authors:** Se-Han Jung, Hong Seon Lee, Min Jung, Kwangho Chung, Hyun-Soo Moon, Sung-Hwan Kim

**Affiliations:** 1 Arthroscopy and Joint Research Institute, Yonsei University College of Medicine, Seoul 03722, Republic of Korea; drshjung@naver.com (S.-H.J.); jmin1103@yuhs.ac (M.J.); khchung85@yuhs.ac (K.C.); oshsdesu@gmail.com (H.-S.M.); 2 Department of Orthopedic Surgery, Severance Hospital, Yonsei University College of Medicine, Seoul 03722, Republic of Korea; 3 Department of Radiology, Gangnam Severance Hospital, Yonsei University College of Medicine, Seoul 06273, Republic of Korea; reanjea55@yuhs.ac; 4 Department of Orthopedic Surgery, Yongin Severance Hospital, Yonsei University College of Medicine, Yongin 06995, Republic of Korea; 5 Department of Orthopedic Surgery, Gangnam Severance Hospital, Yonsei University College of Medicine, Seoul 06273, Republic of Korea

**Keywords:** anterior cruciate ligament, anterior cruciate ligament reconstruction, biologics, biologic augmentation, atelocollagen

## Abstract

*Background and Objectives*: Atelocollagen, a purified collagen derivative, has demonstrated potential benefits in various orthopedic applications; however, its role in anterior cruciate ligament (ACL) reconstruction remains unclear. This study aimed to compare magnetic resonance imaging (MRI) graft signal following ACL reconstruction with and without atelocollagen interposition and to evaluate its effects on knee joint laxity and clinical outcomes. *Materials and Methods*: A retrospective review was conducted on patients who underwent primary ACL reconstruction using hamstring autografts between March 2015 and July 2023. Patients were divided into two groups: without atelocollagen (Group N) and with atelocollagen interposition (Group C). The primary outcome was ACL graft signal intensity (intra-articular and intra-tunnel) on 1-year postoperative MRI. Secondary outcomes included tunnel widening, knee joint laxity, and patient-reported outcome measures (PROMs). *Results*: A total of 57 patients (Group N: 34; Group C: 23) were analyzed. Baseline characteristics were comparable between groups. Group C had a higher proportion of thicker graft constructs (quintuple/sextuple: 73% vs. 17.6%, *p* < 0.001) and more frequent concomitant anterolateral ligament reconstruction (78.3% vs. 8.8%, *p* < 0.001). No significant difference in intra-articular graft signal was observed. However, femoral intra-tunnel graft signal was significantly lower in Group C (*p* = 0.018), accompanied by reduced femoral tunnel widening (*p* < 0.001). Tibial tunnel widening, knee joint laxity, and PROMs did not differ significantly at 1 and 2 years. In multivariable analysis, atelocollagen interposition was associated with reduced femoral tunnel widening (B = −1.1, *p* = 0.025). *Conclusions*: Atelocollagen interposition during ACL reconstruction was associated with more favorable intra-tunnel graft healing signals and reduced femoral tunnel widening, although it did not appear to influence short-term clinical outcomes.

## 1. Introduction

Anterior cruciate ligament (ACL) reconstruction has been the primary surgical treatment for managing ACL injuries and associated knee joint instability, often producing favorable outcomes [1,2,3]. The failure rate after ACL reconstruction varies across studies and patient populations. However, the reported graft re-rupture rate following primary ACL reconstruction ranges from 0% to 13.4%, with clinical failure rates ranging from 1.9% to 25.6%. The overall cumulative ACL failure rate has been reported to range from 3.2% to 27% over follow-up periods exceeding 10 years [4].

To enhance ACL reconstruction outcomes, surgeons have explored various strategies [2,5,6], including biological augmentation during surgery [7,8]. Biological augmentation involves the application of biological substances to promote healing and improve graft incorporation [8]. These substances include biomaterials, growth factors, autologous tissue, and stem cells. However, most studies on biological augmentation remain experimental or have been conducted in animal models, with limited clinical application [8]. Among clinical studies, the use of platelet-rich plasma for augmentation is the most extensively investigated [9,10,11].

Atelocollagen is a purified collagen derivative obtained by removing telopeptide regions from tropocollagen, a soluble collagen molecule that forms collagen fibrils. This process reduces antigenicity while preserving the basic triple-helical collagen structure. Atelocollagen is typically derived from bovine or porcine sources and has been used clinically to support tissue healing [12]. To our knowledge, no studies have explored the direct application of atelocollagen for biological augmentation in ACL reconstruction. However, the use of atelocollagen has been investigated in other orthopedic applications. For instance, in meniscal root repairs, atelocollagen interposition at the repair site has been associated with improved MRI signal in repaired tissue [13]. Similarly, in rotator cuff repairs, atelocollagen patches applied at the attachment site have been reported to be associated with lower re-tear rates [14]. In addition, atelocollagen injection has been associated with improved clinical outcomes in patients with medial collateral ligament rupture compared with those who did not receive atelocollagen injection [15]. Based on these findings, we hypothesized that atelocollagen may have a potential role in supporting intra-tunnel and intra-articular graft healing after ACL reconstruction.

In the present study, type I atelocollagen was interposed within the hamstring autograft composite during ACL reconstruction. Because the technique used in this study involves interposition of atelocollagen between graft strands, it is technically applicable to hamstring autografts, which are typically prepared as multi-stranded tendon constructs. In contrast, this technique is not readily applicable to bone–patellar tendon–bone or quadriceps tendon autografts, which consist of a single tendon component with or without a bone block. Therefore, this study focused on patients who underwent ACL reconstruction using hamstring autografts.

The purpose of this study was to evaluate the effect of atelocollagen interposition within the ACL graft during ACL reconstruction. The primary outcome was graft signal intensity on follow-up MRI, and secondary outcomes included knee joint laxity and clinical outcomes. We hypothesized that patients who received atelocollagen interposition would show lower graft signal intensity on T2-weighted MRI (T2-WI).

## 2. Materials and Methods

Electronic medical record and image data from patients who underwent primary ACL reconstruction by a single senior orthopedic surgeon (S.-H.K.) at a single institution (Gangnam Severance Hospital) between March 2015 and July 2023 were retrospectively reviewed. Inclusion criteria were primary ACL reconstruction using hamstring autografts. Exclusion criteria included: (1) use of alternative graft types (e.g., bone–patellar tendon–bone, quadriceps tendon, or allografts); (2) absence of follow-up MRI at approximately 1 year (minimum follow-up: 1 year); (3) revision ACL reconstruction; (4) staged or concomitant multi-ligament reconstruction, except for anterolateral ligament (ALL) reconstruction; (5) subsequent surgery for complications (e.g., postoperative septic arthritis and large cyclops lesion); (6) contralateral ACL reconstruction; (7) ACL graft re-rupture within 2 years; and (8) physeal-sparing techniques for skeletally immature patients. Because this study aimed to compare 1-year MRI outcomes and postoperative knee joint laxity and clinical outcomes at 1 or 2 years, patients with graft re-rupture within 2 years were excluded, as re-rupture and subsequent revision surgery could confound the assessment of these outcomes. Surgical indications for primary ACL reconstruction included: (1) ACL rupture confirmed on preoperative MRI; (2) significant anterior–posterior instability (Lachman test ≥ grade 2, side-to-side difference [SSD] ≥ 5 mm on KT-2000 or Lachman Telos stress radiographs); (3) significant rotational instability (pivot shift test ≥ grade 2); and (4) combined meniscal tears with complete ACL rupture.

Patients were divided into two groups: Group N (No atelocollagen interposition in hamstring autografts) and Group C (Collagen interposition in hamstring autografts). From April 2022, Type I atelocollagen was interposed within hamstring autografts, wrapped using sutures, to assess its potential to support graft healing. Accordingly, Group C consisted of patients treated during the later practice period. Hamstring autografts were exclusively used, as their multiple bundles allowed efficient wrapping and interposition of atelocollagen. Primary outcomes included intra-articular and intra-tunnel graft signal intensity on 1-year follow-up MRI. Secondary outcomes were tibial and femoral tunnel widenings on 1-year follow-up MRI, knee joint laxity, and PROMs at 1 and 2 years.

### 2.1. Graft Preparation and Interposition of Type 1 Atelocollagen into the ACL Graft

Patients were thoroughly informed about the graft options available for surgery, including bone–patellar tendon–bone autografts, quadriceps tendon autografts, hamstring tendon autografts, and allogeneic tendon grafts. The explanation covered the potential advantages and disadvantages of each option, allowing patients to make an informed decision regarding their preferred graft. This study included only patients who chose a hamstring autograft for their ACL reconstruction.

**Figure 1:**
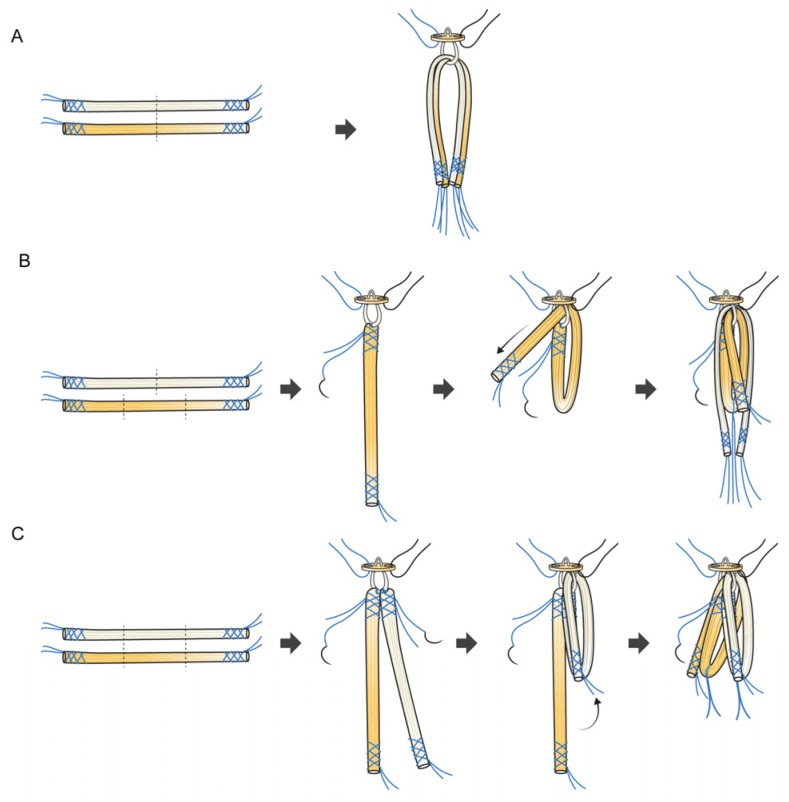
Preparation and configuration of the multiple stranded (quadruple, quintuple, and sextuple) hamstring autograft. (**A**) Quadruple graft preparation. (**B**) Quintuple graft preparation. For the graft intended to be folded into three strands, the thinner end was securely tied to the EndoButton loop, and the opposite end was passed through the loop to create a triple-fold configuration. An additional Ethibond suture was placed at the folded one-third point from the loop to facilitate traction during tensioning. A second tendon was then passed through the loop and folded in half, completing the quintuple graft configuration. (**C**) Sextuple graft preparation. After whipstitching both ends of the tendons, one end of each tendon was securely tied to the EndoButton loop. Each tendon was then passed through the loop to create three folds per tendon. Ethibond sutures were placed at the one-third point from the loop on each tendon to serve as traction sutures, completing the six-stranded graft configuration.

Graft preparation was performed by orthopedic fellows specializing in knee surgery and sports medicine. Atelocollagen interposition was performed by two different orthopedic fellows. The method of graft preparation and atelocollagen interposition was standardized and handed over through joint participation in several surgeries whenever there was a change in personnel, ensuring consistent execution. For the quadruple hamstring graft, both ends of the semitendinosus and gracilis tendons were whipstitched with No. 1 Ethibond sutures (Ethicon Inc. Cincinnati, OH, USA). The tendons were then passed through the loop of the EndoButton CL (Smith&Nephew Inc, Andover, MA, USA), folded in half to form a four-stranded configuration (Figure 1A). For the quintuple graft (five-stranded), the thicker of the two tendons was planned to be folded into three strands, and if the thickness was similar, the longer tendon was prepared as a triple strand. Both tendons were whipstitched at both ends using Ethibond. For the graft intended to be folded into three strands, the thinner end was securely tied to the EndoButton loop to prevent slippage, and the opposite end was passed through the loop to create the triple-fold configuration. An additional Ethibond suture was placed at the folded 1/3 point from the loop to allow for traction during tensioning. A second tendon (usually thinner gracilis tendon) was then passed through the loop and folded in half to complete the quintuple configuration of the graft (Figure 1B). For the sextuple graft, both whipstitched tendons were securely tied to the EndoButton loop at one end. Each tendon was then passed through the loop in the same manner to create three folds per tendon. Ethibond sutures were placed at the 1/3 point from the loop on each tendon to serve as traction sutures. This configuration completed the six-strand tendon graft (Figure 1C).

**Figure 2:**
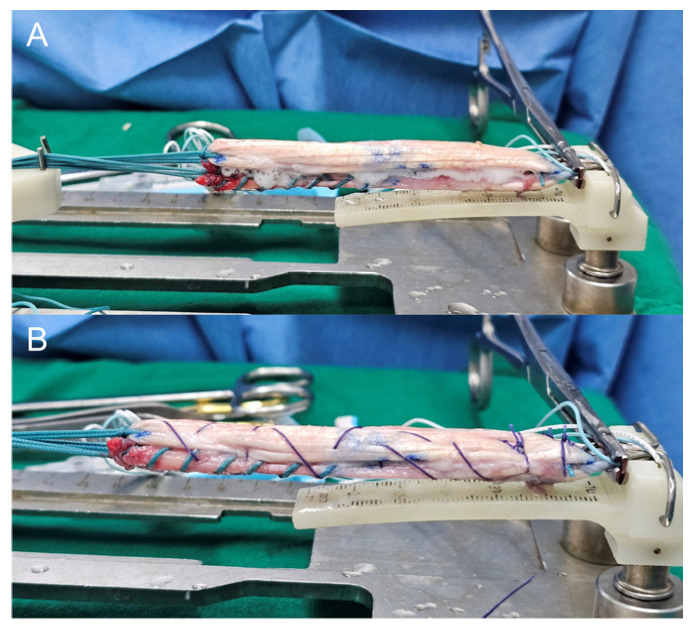
The figures show the process of interposing atelocollagen within a sextuple hamstring graft. (**A**) Atelocollagen was applied between the strands of the sextuple graft. (**B**) Wrapping sutures were applied to prevent the atelocollagen from protruding between the strands.

To interpose collagen within the ACL graft, Type I atelocollagen (3% porcine atelocollagen, COLTRIX® TendoRegen; Ubiosis Co., Ltd., Seongnam, Republic of Korea) was combined with fibrin glue (Greenplast, Green Cross P.D. Co, Yongin, Gyeonggi-do, Republic of Korea) to provide solidity and serve as a scaffold. The atelocollagen–fibrin glue mixture was prepared using two 1 mL syringes and a Y-shaped mixing catheter connected to a 20-gauge needle. One syringe was filled with 1 mL of fibrinogen. The other was filled with 0.9 mL of atelocollagen and 0.1 mL of thrombin. The mixture was applied between the bundles of the prepared hamstring autograft (Figure 2A). After the atelocollagen achieved sufficient solidity, wrapping sutures were applied to prevent collagen extrusion between the bundles (Figure 2B). The prepared ACL graft was then pretensioned using the tensioning device (Graft Master III; Smith&Nephew) at 20 pounds for 20 min.

### 2.2. Surgical Procedures and Rehabilitation

All procedures were performed by a single senior surgeon. The hamstring autograft was harvested before arthroscopic procedures. A vertical incision, approximately 5 cm in length, was made on the anteromedial aspect of the proximal tibia above the pes anserinus to expose the semitendinosus and gracilis tendons near their distal attachments. Accessory bands of the tendons were excised. The tendons were separated to the proximal musculotendinous junction using an open-loop tendon stripper and detached from their tibial insertion. The harvested grafts were then handed for graft preparation.

Diagnostic arthroscopy was performed via a parapatellar high anterolateral portal to evaluate the knee compartments. Meniscal lesions, if present, were treated based on their characteristics prior to ACL reconstruction. The tibial tunnel was drilled at the center of the ACL footprint, with its diameter matched to the graft size. A far anteromedial portal, positioned just above the medial meniscus, was used to create the femoral tunnel. The femoral tunnel was drilled at the ACL femoral footprint with the knee flexed to approximately 120° to ensure sufficient tunnel length and minimize wall compromise. Reaming with the diameter matched to the graft size was performed up to 20 mm in depth. A 4.5 mm reamer was then used to extend the femoral socket to the far cortex, allowing the passage of the EndoButton CL (Smith&Nephew Inc, Andover, MA, USA). The femoral tunnel reaming was completed in appropriate depth, considering the total tunnel length to the far cortex, the graft insertion length, and the loop length of the EndoButton CL. The graft was introduced into the femoral tunnel using an eyelet pin loaded with sutures, and the proper flipping of the EndoButton CL was confirmed arthroscopically to ensure secure cortical suspensory fixation. Distal graft tensioning was performed using a tensioning device (ConMed Linvatec, Largo, FL, USA) approximately 10 N of tension per 1 mm of graft diameter, with a total force of 70–100 N depending on the graft diameter. Cyclic loading (20 cycles) was applied to ensure proper seating. Final tibial tunnel fixation was achieved using a bioabsorbable interference screw (ConMed Linvatec) at a knee flexion angle of approximately 15°, with additional stabilization provided by a cortical screw and washer placed below the tibial tunnel. The surgical technique for combined anterolateral ligament reconstruction is provided in Appendix A.

Postoperative management included controlled range of motion using a hinged knee brace, crutch-assisted ambulation, and partial weight-bearing for the first six weeks. Early rehabilitation focused on restoring knee range of motion and quadriceps isometric strength. During the first two weeks, the knee was maintained in full extension. After two weeks, patients initiated a range of motion from 0–60°, increasing by 30° every two weeks. The rehabilitation protocol was not modified according to whether concomitant meniscal repair was performed. By six weeks, crutches and braces were discontinued, and patients progressed to full weight-bearing ambulation and closed kinetic chain exercises. At six months, open kinetic chain exercises, jogging, and swimming were introduced. Sports activities involving pivoting, jumping, or side-stepping were permitted after nine months.

### 2.3. Assessment of ACL Reconstruction Surgery

**Figure A1:**
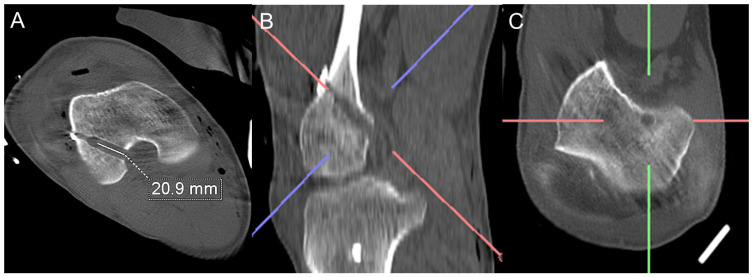
Measurement of graft insertion length (GIL). This is an immediate postoperative CT scan following ACL reconstruction in a 20-year-old male patient. Using MPR, oblique axial (**A**), oblique sagittal (**B**), and oblique coronal (**C**) images were obtained, aligned along the longitudinal axis of the femoral tunnel. In the oblique axial image (**A**), the graft insertion length was measured, excluding the tunnel root height.

Information data about the surgery were collected on the presence and type of combined meniscal surgery, anterolateral ligament reconstruction, and the number of graft bundles used. Immediate postoperative computed tomography (CT) scans (Sensation 64, Siemens Healthcare, Erlangen, Germany) were performed with patients’ consent. Tunnel diameter, tunnel position, and graft insertion length were evaluated. The positions of the femoral and tibial tunnels, as well as the graft insertion length (Figure A1), were measured using methods previously reported in the literature [16,17].

### 2.4. MRI Evaluation (Intra-Articular and Intra-Tunnel Graft Signal, Femoral and Tibial Tunnel Diameter)

**Figure 3:**
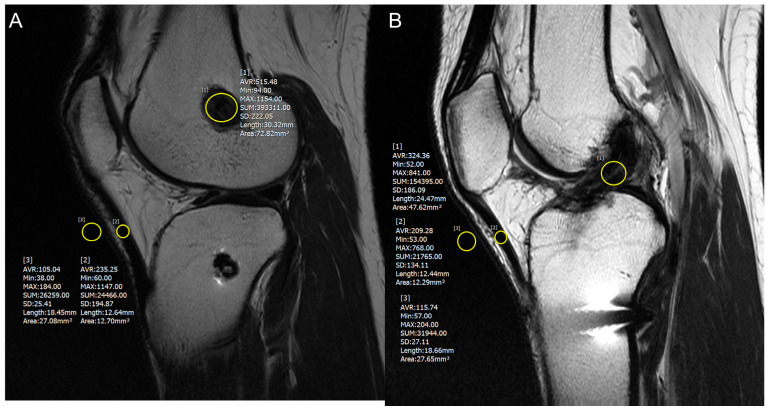
This is an 18-month follow-up MRI of a 42-year-old female patient after ACL reconstruction. Image (**A**) shows the measurement of the contrast-to-noise ratio (CNR) of the intra-tunnel ACL graft, while image (**B**) depicts the measurement of the CNR of the intra-articular graft. To measure the CNR of the intra-tunnel ACL graft, the region of interest (ROI) was drawn as large as possible at the most widened portion of the graft. For the CNR measurement of the intra-articular ACL graft, a circular ROI was centered on the middle third of the ACL graft, with its center aligned with the ACL’s anterior and posterior borders as its diameter. The ROI for the patellar tendon was drawn at its mid-portion, ensuring it was as large as possible while closely aligning with the anterior and posterior margins. Additionally, a background ROI was measured approximately 2 cm anterior to the patellar tendon on its anterior aspect.

**Figure 4:**
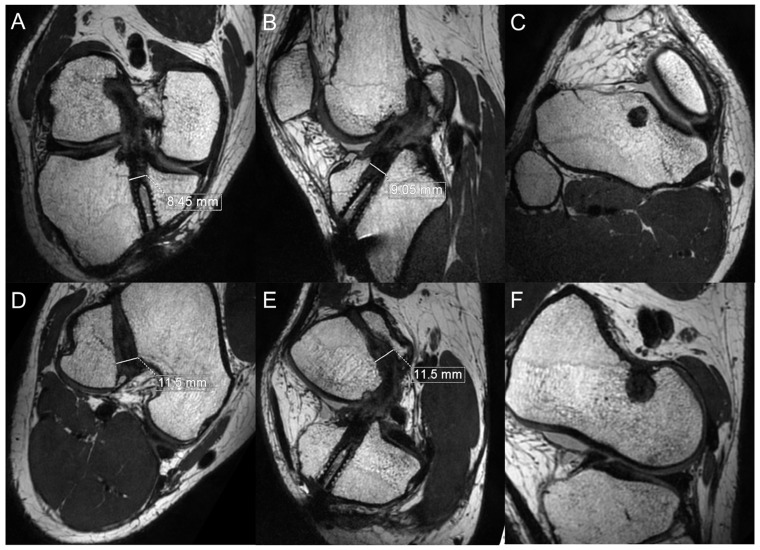
A follow-up study was conducted 12 months after ACL reconstruction in an 18-year-old male patient. Panels (**A**–**C**) are MPR images aligned along the longitudinal axis of the tibial tunnel, while panels (**D**–**F**) are MPR images aligned along the longitudinal axis of the femoral tunnel. For each tunnel, the oblique coronal (**A**,**D**) and oblique sagittal (**B**,**E**) images aligned with the longitudinal axis were used to measure the diameter of the most widened portion. The larger of the two measured values was used for analysis.

Postoperative MRI was performed approximately 1 year after surgery using a 3.0 T GE Discovery MR750 system (GE Healthcare, Waukesha, WI, USA) with an 8-channel knee coil with patient consent. Sagittal T2-weighted images were acquired with the following parameters: slice thickness, 3 mm; echo time, 80 ms; repetition time, 3000 ms; matrix, 416 × 256; field of view, 160 × 160 mm; and no fat suppression. MRI evaluation included the intra-articular and intra-tunnel graft CNR [18], as well as measurements of femoral and tibial tunnel diameters. The CNR of the ACL tibial tunnel was not measured due to artifacts caused by the bioabsorbable interference screw inserted during the surgery. CNR was calculated by selecting a region of interest (ROI) for the graft signal, subtracting the patellar tendon signal, and dividing by the standard deviation of the background signal. For intra-articular ACL grafts, measurements were obtained on sagittal T2-weighted imaging (T2WI), focusing on the mid-third portion of the graft. To measure the CNR of the intra-articular ACL graft, a circular ROI was centered on the mid-third portion, with its center aligned with the ACL’s anterior and posterior borders as its diameter. Intra-tunnel graft CNR was measured at the femoral tunnel, ensuring the absence of artifacts and selecting the sagittal T2WI slice where the tunnel appeared most widened (Figure 3). Femoral and tibial tunnel diameters were measured using MPR generated from a 3D non-fat-suppressed sequence, with the diameter recorded at the slice showing the largest tunnel dimensions (Figure 4). We used Aquaris Intuition Edition ver 4.4.13.p6A (Aquiris Medical Solutions, Zurich, Switzerland) to obtain MPR images. Tunnel widening was calculated by subtracting the initial tunnel diameter from the tunnel diameter measured on follow-up MRI. ROI and tunnel diameter measurements were performed by a musculoskeletal radiologist with 7 years of experience, who was blinded to treatment group, graft configuration, concomitant ALL reconstruction, and clinical outcomes.

### 2.5. Clinical Outcome Assessment

Physical examination using Lachman and pivot shift test was performed to evaluate the instability pre- and postoperatively. AP laxity was measured by SSD using KT-2000 arthrometer and Lachman Telos stress radiographs pre- and postoperatively. Postoperative objective clinical outcome was assessed with International Knee Documentation Committee (IKDC) examination form (final grade A/B/C/D). AP laxity section of the IKDC examination form was assessed based on the results of the KT-2000 arthrometer. Physical examinations and KT-2000 arthrometer test were performed once by the senior orthopedic surgeon during the patient’s visit to the out-patient department. Radiographic data such as SSD from Lachman Telos Stress radiograph was measured twice by one orthopedic surgeon specializing in sports medicine and one musculoskeletal radiologist with at least one month interval. Postoperative outcomes were evaluated at 1-year and 2-year follow-up, respectively. Subjective clinical outcome was assessed with several PROMs, including pain visual analogue scale (VAS), Lysholm knee score, IKDC subjective score, and Knee injury and Osteoarthritis Outcome Score (KOOS). PROMs were collected through face-to-face interviews conducted by an orthopedic research assistant preoperatively and one and two years postoperatively.

### 2.6. Statistical Method

All statistical analyses were performed using SPSS version 26.0 (IBM, Armonk, NY, USA), with statistical significance defined as *p* < 0.05. Values are presented as mean ± standard deviation unless otherwise indicated. Continuous variables between the two groups were compared using an independent t-test or Mann–Whitney U test depending on the normality test (Shapiro–Wilk test). Categorical variables from the two groups were compared primarily using chi-squared tests. Fisher’s exact test was applied when there was one or more cells with an expected frequency of 5 or less. For between-group comparisons of clinical outcomes at the final follow-up, the most recent available postoperative data for each patient were used, regardless of whether the patient completed the 2-year follow-up.

Subgroup analysis was conducted to evaluate factors influencing the outcomes. Subgroups were divided based on whether ALL reconstruction was performed and whether quintuple or sextuple hamstring grafts were used, as these were considered potential main confounding factors. The analytic methods were consistent with those used for comparisons between the main groups. To analyze the relationship between femoral tunnel widening and various factors, Spearman’s test was primarily used. Additionally, multiple regression analysis was performed to identify factors significantly influencing ACL tunnel widening. The variance inflation factor (VIF) was used to assess multicollinearity among the variables included in the analysis. To present the intra- and inter-observer reliability of the measurement methods used in the analysis, intraclass correlation coefficient was employed. Statistical power analysis for the significant results was conducted using G*Power (ver. 3.1, University Düsseldorf, Düsseldorf, Germany).

## 3. Results

**Figure 5:**
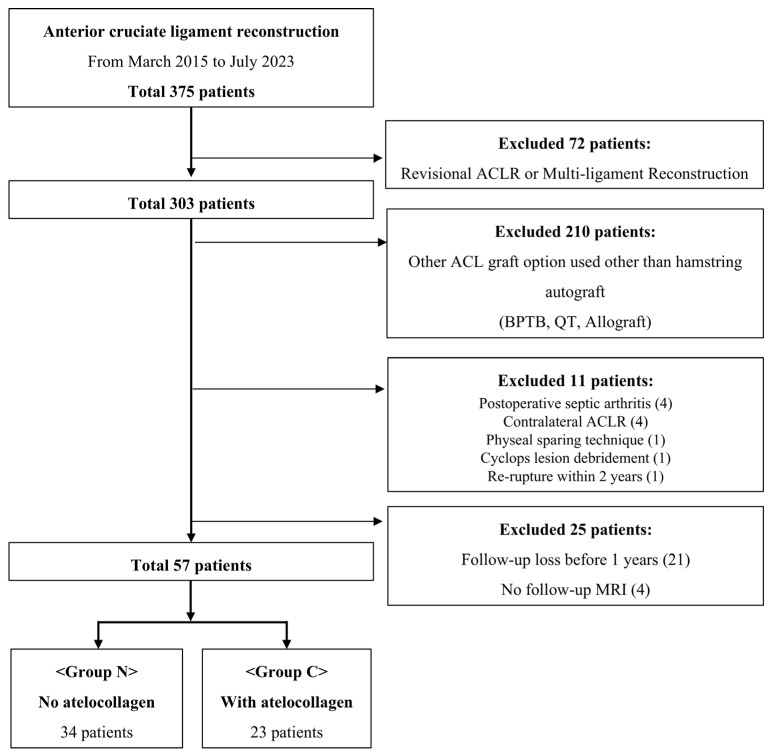
Patient flow diagram. ACLR, anterior cruciate ligament reconstruction; ACL, anterior cruciate ligament; BPTB, bone–patellar tendon–bone graft; QT, quadriceps tendon; MRI, magnetic resonance imaging. Exclusion criteria were applied sequentially in the order shown in the flow diagram. Patients included in each exclusion box met only the corresponding exclusion criterion and were not counted in multiple exclusion categories.

A total of 57 patients were included in the study: 34 patients in Group N (No collagen) and 23 in Group C (Collagen) (Figure 5). Demographic characteristics and preoperative instability parameters are presented in Table 1 and did not show differences between the groups. The surgical details for the included patients are also presented in Table 1. The diameter of the ACL femoral and tibial tunnel was significantly wider in group C compared to group N, as the grafts in group C were thicker due to the use of quintuple and sextuple grafts rather than the quadruple hamstring grafts used in group N. Anterolateral ligament (ALL) reconstruction was performed in Group C, as incorporating ALL reconstruction and atelocollagen interposition in primary ACL reconstruction has been considered in more recent surgical practices. Other factors showed no differences between the two groups.

### 3.1. MRI Assessments

MRI was performed on the included patients at a mean of 12.7 months postoperatively. There was no significant difference in intra-articular ACL graft signal (CNR, contrast-to-noise ratio) between the groups (Table 2). However, intra-tunnel ACL graft signal at the femoral tunnel was significantly lower in group C than group N. Additionally, femoral widening measured on multiplanar reconstruction (MPR) MRI was significantly narrower in group C than in group N, while tibial tunnel widening showed no significant difference between the groups. A post hoc power analysis based on the observed effect sizes demonstrated that the statistical power for the statistically significant primary outcome measures ranged from 66.1% to 95.3%. The ICC values for the MRI assessments ranged from 0.973 to 0.990 for intra-observer reliability and 0.882 to 0.946 for inter-observer reliability, indicating good to excellent reliability.

### 3.2. Knee Instability and Objective/Subjective Clinical Outcomes

**Figure 6:**
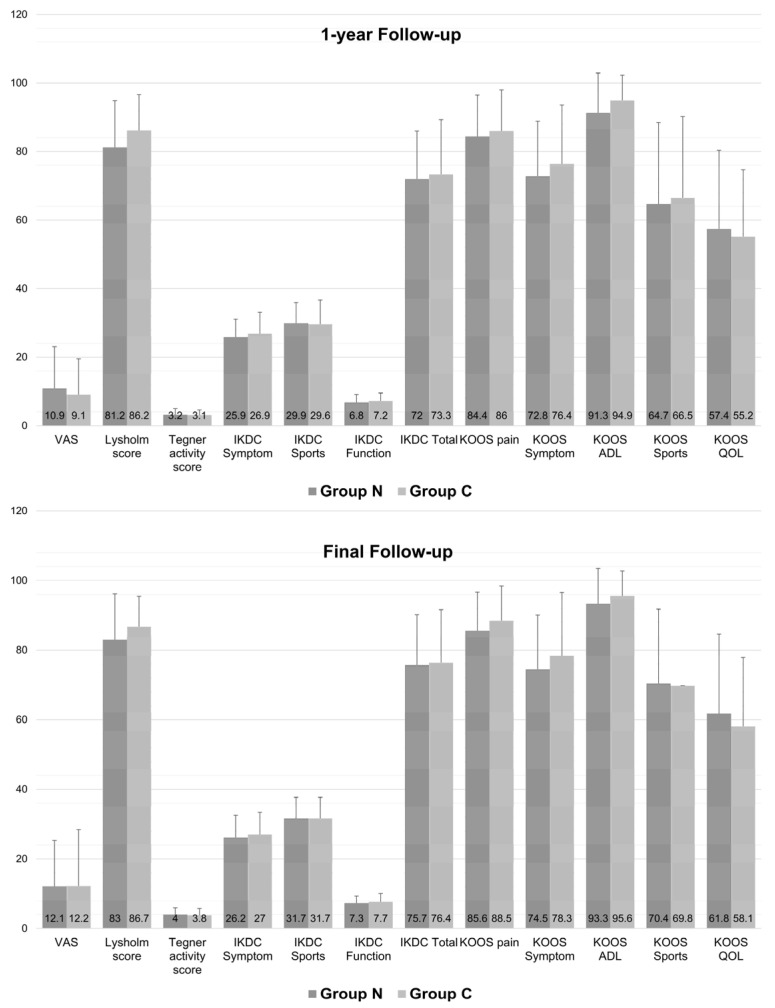
Patient-reported outcomes at 1-year follow-up and final follow-up. Final follow-up values were based on the most recent available data for each patient. The 2-year loss-to-follow-up rates were 23.5% in Group N and 26.1% in Group C, with mean final follow-up periods of 21.8 and 21.5 months, respectively. Error bars indicate standard deviations. Visual analogue scale, VAS; IKDC, International Knee Documentation Committee; KOOS, Knee injury and Osteoarthritis Outcome Score; ADL, activity of daily living; QOL, quality of life.

At the 1-year postoperative and the final follow-up, comparisons of various instability-related parameters between the two groups showed no significant differences (Table 3). No difference was found in PROMs (Figure 6). At the 2-year follow-up, some patients were lost to follow-up. For the final follow-up analysis of clinical outcomes, the most recent available follow-up data were used for each patient, including 1-year data for patients who did not complete the 2-year follow-up. The 2-year loss to follow-up rates were 8/34 (23.5%) in group N and 6/23 (26.1%) in group C. The mean final follow-up period was approximately 21.8 months in group N and 21.5 months in group C.

### 3.3. Subgroup Analysis (ALL Reconstruction, Quintuple or Sextuple Graft)

The entire cohort was subdivided based on whether ALL reconstruction was performed and whether quintuple or sextuple grafts were used, and similar comparative analyses were subsequently performed. The results revealed a pattern consistent with the comparisons between the main groups. While knee joint instability and clinical outcomes at the final follow-up showed no significant differences between subgroups, the degree of femoral tunnel widening and intra-tunnel graft signal (femoral) demonstrated significant differences (Table A1 and Table A2). Femoral tunnel widening was smaller in the ALL reconstruction group and quintuple/sextuple group compared to the non-ALL reconstruction group and quadruple graft group (ALL vs. non-ALL reconstruction group, 1.5 ± 1.3 mm vs. 2.3 ± 1.3 mm, *p* = 0.026; quintuple/sextuple vs. quadruple graft group, 1.3 ± 1.3 mm vs. 2.4 ± 1.2 mm, *p* < 0.001). The intra-femoral tunnel CNR was also significantly lower in the ALL reconstruction group and quintuple/sextuple graft group compared to the non-ALL reconstruction group and quadruple group (ALL vs. non-ALL reconstruction group, 19.4 ± 14.5 vs. 9.7 ± 5.3, *p* < 0.001; quadruple vs. quintuple/sextuple graft group, 18.9 ± 13.7 vs. 11.4 ± 9.9, *p* = 0.020). Additionally, tibial tunnel widening was significantly smaller in the quintuple/sextuple graft group compared to the quadruple group (quadruple vs. quintuple/sextuple graft group, 1.6 ± 1.0 mm vs. 0.7 ± 1.8 mm, *p* = 0.028).

### 3.4. Factors Associated with Tunnel Widening (Spearman’s Test and Multiple Regression Analysis)

Atelocollagen interposition, ALL reconstruction, and the use of quintuple/sextuple grafts were all significantly associated with femoral tunnel widening in Spearman’s test. Tibial tunnel widening was significantly associated only with the use of quintuple or sextuple grafts (Table 4). In the multiple regression analysis with femoral tunnel widening as the dependent variable, atelocollagen interposition was the only factor significantly associated with femoral tunnel widening (B = −1.111, 95% CI, −2.074 to −0.148; *p* = 0.025; VIF = 2.286). Quintuple/sextuple graft use (B = −0.639, 95% CI, −1.418 to 0.140; *p* = 0.106; VIF = 1.496) and ALL reconstruction (B = −0.305, 95% CI, −1.229 to 0.620; *p* = 0.512; VIF = 2.039) were not significantly associated with femoral tunnel widening.

## 4. Discussion

The principal findings of this study were as follows: (1) In this study, the primary outcome measure, graft signal, was assessed using CNR. The results showed that, in Group C, where atelocollagen interposition was performed, there was no difference in intra-articular graft signal compared to Group N, which did not use atelocollagen. However, the intra-tunnel graft signal showed lower signal intensity on T2-weighted imaging (T2WI) in Group C. (2) An additional important finding from the analysis was that Group C showed significantly less femoral tunnel widening compared to Group N. (3) Femoral tunnel widening may have been influenced not only by atelocollagen interposition but also by the use of quintuple/sextuple thicker grafts and ALL reconstruction. However, multiple regression analysis indicated that femoral tunnel widening was significantly associated with atelocollagen interposition. In contrast, tibial tunnel widening was associated with the use of quintuple/sextuple grafts. (4) Postoperative knee joint instability and PROMs showed no significant differences between the two groups.

Recent advancements in orthopedic surgery have introduced the use of innovative biomaterials to promote tissue healing [19]. One such biomaterial is atelocollagen, a highly purified, cell-free form of type I collagen designed to provide matrix stability and reduce immunogenicity. Atelocollagen is produced by removing the telopeptide regions from collagen, thereby reducing antigenicity and lowering the likelihood of immune reactions [20]. The use of atelocollagen is particularly relevant for tendons or ligaments, which consist largely of type I collagen. Studies have reported the safety and effectiveness of atelocollagen, highlighting its role in improving tissue repair and supporting tendon injury healing [14,15,21,22]. Margarian et al. conducted a safety-focused study in a porcine model, demonstrating that the intra-articular application of atelocollagen during ACL repair did not lead to an increase in local or systemic inflammation [21]. In some animal and clinical studies, collagen has been used to wrap ACL grafts to serve as a scaffold or reservoir for delivering biologics such as platelet-rich plasma or bone marrow aspirate concentrate [23,24]. These studies did not investigate the effects of collagen alone. Clinical studies focusing solely on the use of atelocollagen to enhance ACL reconstruction outcomes are currently limited. In other tendon and ligament pathologies beyond ACL, several studies have reported positive effects associated with the use of atelocollagen. The most extensively studied clinical application of atelocollagen appears to be in the field of rotator cuff tears [14,25,26]. Kim et al. reported that intra-articular injection of atelocollagen in patients with partial-thickness rotator cuff tears led to improvements in functional and pain scores at 12 months, as well as enhanced tendon integrity on MRI at 6 months [25]. Kim et al. have also reported that injection of atelocollagen during rotator cuff repair intra-operatively could enhance radiographic tendon integrity [26]. Atelocollagen is also used in patch type for rotator cuff repairs, and a study found that applying patch-type atelocollagen at the attachment site during rotator cuff tear repair significantly reduced the re-tear rate [14]. Beyond rotator cuff tears, Jang et al. demonstrated that patients with grade 3 MCL injuries who received atelocollagen injections had better clinical outcomes compared to those who did not receive injections [15]. Inserted atelocollagen can be encapsulated or degraded by some immune responses, but it has also been reported to be adopted by fibroblasts, contributing to the formation of new tissue and aiding in tissue healing [27]. In the study by Suh et al., the use of an atelocollagen patch in rotator cuff repair in a rabbit model was reported to promote the remodeling of type III collagen to type I collagen during the final stage of the tendon-to-bone healing process [22]. Immunohistochemistry showed stronger expression of type I collagen in the collagen patch group.

In this study, atelocollagen interposition within the ACL graft did not yield a significant difference in intra-articular graft signal between the two groups. However, intra-tunnel graft signal on T2WI was significantly lower in Group C. This suggests that, in the intra-articular portion, interposed atelocollagen may have been more vulnerable to degradation and resorption. In contrast, intra-tunnel collagen likely persisted longer, contributing to graft healing and fibrous tissue generation. Additionally, femoral tunnel widening was significantly smaller in Group C. This may also be related to the specific actions of atelocollagen within the graft. All patients in our study used hamstring autografts with suspensory fixation on the femoral side, which has been associated with a higher risk of tunnel widening due to the “windshield wiper” and “bungee cord” effects [28,29,30,31]. The reduction in femoral tunnel widening with atelocollagen interposition may be related to the mitigation of these effects. We hypothesize that atelocollagen interdigitated between the graft strands may contribute to strand-to-strand incorporation, fibrous tissue formation, and tendon-to-bone healing, thereby reducing micromotion within the tunnel and minimizing tunnel widening.

Femoral tunnel widening may have been influenced not only by the use of atelocollagen but also by the use of thicker quintuple or sextuple grafts and concurrent ALL reconstruction. This is because Group C had significantly higher rates of both quintuple/sextuple graft use and combined ALL reconstruction compared to Group N. To identify the factors associated with femoral tunnel widening, multiple regression analysis was performed, which showed that the use of atelocollagen was the only significant factor. However, this result should be interpreted cautiously, as the possibility of selection bias and confounding by surgical factors cannot be excluded. Although quintuple/sextuple graft use and ALL reconstruction were not significantly associated with femoral tunnel widening in this multiple regression analysis, these findings do not necessarily indicate that these factors had no effect. The more frequent use of quintuple/sextuple grafts in Group C resulted in larger graft diameters than in Group N, and graft configuration may have contributed to tunnel-related changes, as suggested by its significant association with tibial tunnel widening. In addition, ALL reconstruction may have affected tunnel widening through its influence on rotational stability, although this was not confirmed statistically in the present study. Therefore, the association between atelocollagen interposition and reduced femoral tunnel widening should be considered exploratory rather than definitive evidence of an isolated causal effect [32,33]. In studies comparing quintuple hamstring autografts with quadruple grafts, results indicated that, for grafts of the same diameter, short-term outcomes were comparable, emphasizing the direct relationship between graft size and surgical outcomes [34,35]. In our study, the more frequent use of quintuple/sextuple grafts in Group C resulted in thicker grafts compared to Group N, which may have contributed to the observed differences in femoral tunnel widening. Although quintuple/sextuple grafts did not show statistical significance in the multiple regression analysis of this study, their contribution cannot be completely ruled out. Interestingly, this study did find a significant association between the use of quintuple/sextuple grafts and tibial tunnel widening. Furthermore, since ALL reconstruction is known to significantly reduce rotational instability following ACL reconstruction [36,37,38], it is plausible to hypothesize that this additional procedure could also play a role in minimizing tunnel widening.

In terms of subjective clinical outcomes (PROM) and knee joint stability parameters, no significant differences were observed between the two groups. This may be due to the short-term follow-up period of the study. Structural differences do not always translate directly into clinical outcomes, and long-term follow-up is often required. Therefore, this study was designed to primarily assess graft signal differences on MRI.

In summary, our findings suggest that interposing atelocollagen within the ACL graft was associated with lower intra-tunnel graft signal intensity and reduced femoral tunnel widening. However, these findings should be interpreted cautiously, and further studies are needed to determine whether atelocollagen interposition has a clinically meaningful augmentative effect.

This study has several limitations. First, the retrospective nature of the analysis may have introduced inherent biases, as patient selection and data collection were not controlled prospectively. Second, the relatively small sample size could limit the generalizability of our findings and reduce the statistical power to detect subtle differences between groups. Third, the potential for selection bias cannot be overlooked, as the study population may not be fully representative of the broader patient cohort. Fourth, confounding factors that were not accounted for may have influenced our results, given the observational design of the study. Although multiple regression analysis was performed to adjust for potential confounders, the model should be interpreted as exploratory rather than confirmatory because of the limited sample size and the strong imbalance among atelocollagen use, graft strand number, ALL reconstruction, and surgical era. Additional sensitivity analyses including variables such as initial femoral tunnel diameter, graft insertion length, BMI, and sex would have strengthened the analysis; however, further model expansion was limited by the small sample size and the potential risk of overfitting. Therefore, residual confounding cannot be excluded, and the present findings should not be interpreted as evidence of a definitive causal effect of atelocollagen interposition. Additionally, the relatively short-term MRI follow-up period may have restricted our ability to assess long-term changes or outcomes. Finally, the limited correlation with clinical outcomes or PROMs reduces the ability to draw definitive conclusions regarding the clinical significance of our imaging-based findings.

### Clinical Implication

Atelocollagen interposition within the ACL graft was associated with more favorable intra-tunnel graft signal intensity on MRI and reduced femoral tunnel widening. These findings suggest that atelocollagen interposition may have a supportive role in ACL graft healing; however, they should be interpreted as exploratory. Further prospective randomized studies are needed to confirm these findings, and long-term follow-up is required to determine whether these imaging findings translate into meaningful clinical benefits.

## 5. Conclusions

Atelocollagen interposition during ACL reconstruction was associated with more favorable intra-tunnel graft healing signals and reduced femoral tunnel widening, although it did not appear to influence short-term clinical outcomes.

## Figures and Tables

**Table 1:** Demographic characteristics, preoperative instability, and ACL reconstruction surgery details.

Variables	Group N N = 34	Group C N = 23	*p*-Value
Demographic characteristics			
Age	31.2 ± 11.7	31.1 ± 9.8	0.960
Sex, male/ female	30/4	17/6	0.163
Body mass index	25.1 ± 3.3	23.7 ± 2.8	0.053
Affected side, right/left	22/12	9/14	0.057
Preoperative instability			
Lachman test, grade 0/1/2/3	0/7/27/0	0/2/20/1	0.246
Pivot shift test, grade 0/1/2/3	1/15/12/6	0/4/13/6	0.139
Anterior instability (KT-2000), mm	6.3 ± 1.7	6.7 ± 1.7	0.369
ACL reconstruction surgery details			
ACL femoral tunnel diameter	7.8 ± 0.7	8.3 ± 0.6	0.009 *
ACL tibial tunnel diameter	8.2 ± 0.7	8.8 ± 0.5	<0.001 *
Hamstring autograft			<0.001 *
Quadruple	28	6	
Quintuple	6	13	
Sextuple	0	4	
ACL femoral tunnel position			
Depth, %	29.6 ± 5.7	28.2 ± 5.2	0.388
Height, %	30.0 ± 6.7	30.0 ± 6.4	0.974
ACL tibial tunnel position			
Depth, %	33.5 ± 4.2	34.6 ± 4.4	0.376
Width, %	44.7 ± 1.7	43.3 ± 3.2	0.067
Graft insertion length	19.8 ± 3.9	21.1 ± 2.4	0.167
Combined ALL reconstruction	3 (8.8%)	18 (78.3%)	<0.001 *
Medial meniscus procedure			0.367
None	24	12	
Partial meniscectomy	1	1	
Subtotal meniscectomy	0	0	
Repair	9	10	
Lateral meniscus procedure			0.626
None	26	16	
Partial meniscectomy	1	2	
Subtotal meniscectomy	1	0	
Repair	6	5	

ACL, anterior cruciate ligament; ALL, anterolateral ligament. * Statistically significant.

**Table 2:** ACL ligament and intra-tunnel signal intensity assessments and tunnel diameters on 1-year MRI.

	Group N N = 34	Group C N = 23	*p*-Value
Intra-articular graft CNR	12.8 ± 10.2	11.9 ± 14.6	0.105
Intra-tunnel graft CNR (femoral)	19.1 ± 13.6	11.1 ± 9.9	0.018 *
Follow-up femoral tunnel diameter	10.3 ± 1.4	9.5 ± 1.3	0.039
Femoral tunnel widening	2.4 ± 1.2	1.2 ± 1.2	<0.001 *
Femoral tunnel widening (%)	32.1 ± 15.6	14.9 ± 15.0	<0.001 *
Follow-up tibial tunnel diameter	9.7 ± 1.1	9.7 ± 1.9	0.843
Tibial tunnel widening	1.5 ± 1.0	1.0 ± 1.9	0.208
Tibial tunnel widening (%)	18.1 ± 12.6	11.2 ± 22.0	0.141

Postoperative MRI taken at mean 12.7 months post-surgery. Tunnel widening was calculated by subtracting the initial tunnel diameter from the tunnel diameter measured on follow-up MRI. ACL, anterior cruciate ligament; MRI, magnetic resonance imaging; CNR, contrast-to-noise ratio. * Statistically significant.

**Table 3:** Postoperative 1-year and final follow-up instability and objective clinical outcome.

Variables	1-Year Follow-Up			Final Follow-Up		
Group	Group N N = 34	Group C N = 23	*p*-Value	Group N N = 34	Group C N = 23	*p*-Value
SSD (KT-2000)	0.8 ± 1.6	1.3 ± 2.0	0.242	1.3 ± 1.9	1.5 ± 1.9	0.805
SSD (Lachman telos stress view)	2.0 ± 2.7	2.1 ± 2.3	0.929	2.3 ± 3.0	2.0 ± 2.7	0.748
Lachman test, G0/1/2/3	28/6/0/0	16/7/0/0	0.259	24/10/0/0	15/8/0/0	0.669
Pivot test, G0/1/2/3	32/2/0/0	21/2/0/0	0.683	31/3/0/0	20/3/0/0	0.611
IKDC grade, A/B/C/D	20/13/1/0	13/8/2/0	0.631	21/12/1/0	13/9/1/0	0.907

SSD, side-to-side difference; IKDC, International Knee Documentation Committee.

**Table 4:** Factors associated with tunnel widening.

	Femoral Tunnel Widening		Tibial Tunnel Widening	
	Coefficient *r*	*p*-Value	Coefficient *r*	*p*-Value
Atelocollagen interpositi on	−0.510	<0.001 *	-0.151	0.262
Quintuple/ sextuple graft use	−0.467	<0.001 *	-0.316	0.017 *
ALL reconstruction	−0.312	<0.018 *	-0.052	0.701

ALL, anterolateral ligament. * Statistically significant.

## Data Availability

The datasets generated and/or analyzed during the current study are not publicly available due to privacy and ethical restrictions but are available from the corresponding author upon reasonable requests.

## Abbreviations

The following abbreviations are used in this manuscript:

ACL	Anterior Cruciate Ligament
ACLR	Anterior Cruciate Ligament Reconstruction
ALL	Anterolateral Ligament
MRI	Magnetic Resonance Imaging
T2WI	T2-Weighted Imaging
CT	Computed Tomography
CNR	Contrast-to-Noise Ratio
ROI	Region of Interest
MPR	Multiplanar Reconstruction
PROMs	Patient-Reported Outcome Measures
VAS	Visual Analogue Scale
IKDC	International Knee Documentation Committee
KOOS	Knee Injury and Osteoarthritis Outcome Score
SSD	Side-to-Side Difference
AP	Anteroposterior
VIF	Variance Inflation Factor
ICC	Intraclass Correlation Coefficient
ADL	Activities of Daily Living
QOL	Quality of Life

## Appendix A

### Anterolateral Ligament Reconstruction Technique

Tibialis anterior allografts were used for ALL reconstruction. The allograft was trimmed to pass through the 6 mm diameter tunnels. Both ends of the ALL graft were whipstitched using No. 2 Ethibond, and the graft diameter was measured using a slot-sizing block. ALL reconstruction was performed before ACL graft fixation when combined with ACL reconstruction. For anatomical ALL reconstruction, three key landmarks were marked: the lateral femoral epicondyle (LFE), fibular head, and Gerdy’s tubercle. A 20 mm incision was made at the LFE, followed by longitudinal division of the iliotibial band. The femoral attachment site, located slightly proximal and posterior to the LFE, was marked. A guide pin was inserted distally and slightly anteriorly from the femoral attachment site and drilled to a depth of about 30 mm for suture anchor fixation. The femoral side of the ALL tendon was then fixed using a Swivelock suture anchor (Arthrex, Inc., Naples, FL, USA). A 20 mm incision was made between Gerdy’s tubercle and the fibular head, approximately 10 mm below the joint line, to approach the tibial attachment site of the ALL graft. The tibial attachment site was marked and, to avoid collision with the pre-drilled ACL tibial tunnel, the tibial guide pin was directed approximately 30° distally, passing through to the far cortex. Reaming was performed along the guide pin to a depth of 30–35 mm with a 6 mm diameter drill. The whipstitched suture on the tendon was passed through the tibial tunnel along the guide pin to the opposite side. The suture was pulled to pass the ALL graft through the tibial tunnel. Tension was applied to the graft strands while assessing the anisometry of the ALL graft (tighter in extension and lax in flexion), and cyclic tensioning was performed. Finally, the graft was fixed using a bioabsorbable interference screw (ConMed Linvatec) at approximately 15° of knee flexion and neutral rotation.

**Table A1:** Subgroup analysis (no ALL reconstruction vs. ALL reconstruction group).

	No ALLR (N = 36)	ALLR (N = 21)	*p*-Value
Intra-tunnel graft CNR	19.4 ± 14.5	9.7 ± 5.3	<0.001 *
Intra-articular graft CNR	14.1 ± 12.6	9.6 ± 10.9	0.178
1-year femoral tunnel diameter	10.2 ± 1.4	9.7 ± 1.3	0.237
Femoral tunnel widening	2.3 ± 1.3	1.5 ± 1.3	0.026 *
1-year tibial tunnel diameter	9.5 ± 1.7	10.0 ± 0.9	0.132
Tibial tunnel widening	1.2 ± 1.7	1.4 ± 1.7	0.544
Anterior laxity (KT-2000)	1.4 ± 1.7	1.4 ± 2.1	0.963
Anterior laxity (Lachman telos stress view)	1.7 ± 1.4	2.3 ± 3.5	0.429
Lachman test, G0/1/2/3	26/10/0/0	13/8/0/0	0.419
Pivot test, G0/1/2/3	33/3/0/0	18/3/0/0	0.659
IKDC grade, A/B/C/D	24/11/1/0	12/8/1/0	0.754
VAS	9.4 ± 10.4	17.1 ± 18.7	0.094
Lysholm knee score	85.4 ± 11.3	82.6 ± 12.5	0.399
Tegner activity score	4.1 ± 1.9	3.3 ± 1.7	0.097
IKDC Symptom	27.1 ± 6.7	25.1 ± 5.7	0.237
IKDC Sports	32.6 ± 6.0	29.8 ± 6.4	0.104
IKDC Function	7.6 ± 2.0	6.8 ± 2.5	0.145
IKDC Total	78.3 ± 14.5	70.8 ± 14.9	0.070
KOOS Pain	87.6 ± 9.6	85.1 ± 12.4	0.390
KOOS Symptom	76.9 ± 16.0	74.2 ± 18.5	0.561
KOOS ADL	95.1 ± 6.8	91.5 ± 11.9	0.158
KOOS Sports	72.8 ± 20.4	65.2 ± 21.6	0.193
KOOS QOL	62.0 ± 22.9	56.2 ± 19.5	0.340

ALL, anterolateral ligament; ALLR, anterolateral ligament reconstruction; CNR, contrast-to-noise ratio; IKDC, International Knee Documentation Committee; VAS, visual analogue scale; KOOS, Knee injury and Osteoarthritis Outcome score; ADL, activity of daily living; QOL, quality of life.

**Table A2:** Subgroup analysis (quadruple vs. quintuple/sextuple hamstring graft groups).

	Quadruple N = 34	Quintuple/Sextuple N = 23	*p*-Value
Intra-tunnel graft CNR	18.9 ± 13.7	11.4 ± 9.9	0.020 *
Intra-articular graft CNR	13.4 ± 10.0	11.1 ± 14.8	0.484
1-year femoral tunnel diameter	10.3 ± 1.2	9.5 ± 1.5	0.037 *
Femoral tunnel widening	2.4 ± 1.2	1.3 ± 1.3	<0.001 *
1-year tibial tunnel diameter	9.9 ± 1.1	9.4 ± 1.8	0.209
Tibial tunnel widening	1.6 ± 1.0	0.7 ± 1.8	0.028 *
Anterior laxity (KT-2000)	1.3 ± 2.0	1.5 ± 2.2	0.644
Anterior laxity (Lachman telos stress view)	2.2 ± 2.7	1.5 ± 2.2	0.262
Lachman test, G0/1/2/3	24/10/0/0	15/8/0/0	0.669
Pivot test, G0/1/2/3	31/3/0/0	20/3/0/0	0.677
IKDC grade, A/B/C/D	19/13/2/0	17/6/0/0	0.264
VAS	11.8 ± 13.6	13.0 ± 15.8	0.745
Lysholm knee score	84.6 ± 11.8	84.0 ± 11.9	0.872
Tegner activity score	4.2 ± 1.8	3.3 ± 1.8	0.061
IKDC Symptom	26.2 ± 6.8	26.6 ± 5.8	0.850
IKDC Sports	31.3 ± 6.6	32.0 ± 5.6	0.696
IKDC Function	7.1 ± 2.4	7.7 ± 1.8	0.283
IKDC Total	75.1 ± 16.1	76.1 ± 13.4	0.810
KOOS pain	86.3 ± 9.9	87.2 ± 11.9	0.759
KOOS Symptom	73.9 ± 17.4	78.7 ± 16.0	0.298
KOOS ADL	93.5 ± 7.6	94.1 ± 11.0	0.805
KOOS Sports	69.6 ± 22.2	70.7 ± 19.4	0.849
KOOS QOL	60.7 ± 24.2	58.7 ± 17.9	0.739

CNR, contrast-to-noise ratio; IKDC, International Knee Documentation Committee; VAS, visual analogue scale; KOOS, Knee injury and Osteoarthritis Outcome score; ADL, activity of daily living; QOL, quality of life.
